# Validation of a Scale on Society’s Attitudes towards the Sexuality of Women with Intellectual Disabilities—Survey Study

**DOI:** 10.3390/ijerph192013228

**Published:** 2022-10-14

**Authors:** Mónica Rojas-Chaves, Manuel Lucas-Matheu, Gracia Castro-Luna, Tesifón Parrón-Carreño, Bruno José Nievas-Soriano

**Affiliations:** Nursing, Physiotherapy, and Medicine Department, University of Almería, 04120 Almería, Spain

**Keywords:** sexuality, intellectual disability, questionnaire, scale, validation

## Abstract

Background: The main aim of this study was to design and validate a questionnaire in Spanish to acknowledge the perception of the sexuality of women with intellectual disabilities. We sought to cover specific spheres of sexuality, such as sexual capacity, decision-making capacity, sexual disinhibition, and sexual education and quality of life. Methods: A questionnaire was developed based on a literature review. Validation was performed using content validation through a panel of experts; construct validation was performed using exploratory and confirmatory factorial analyses; reliability tests were also performed, using Cronbach’s Alpha and the two-halves test. Results: Two-hundred forty-four participants pilot tested the initial 34-item questionnaire. After content validation and exploratory factorial analysis, the resulting 10-item questionnaire showed four domains, with Cronbach’s Alpha values between 0.69 and 0.82. Confirmatory factorial analysis confirmed the domains, and the model’s goodness-of-fit tests were adequate. Conclusions. The final ten-item scale developed in this research proved to be a valid and reliable instrument, as it has good psychometric properties of both validity and reliability. Thus, researchers interested in investigating the social perception of the sexuality of women with intellectual disabilities can use this tool. Future research can extend the validity of this scale to other languages and settings.

## 1. Introduction

Sexuality is an essential aspect of life [1]. It encompasses sex, gender identities and roles, sexual orientation, eroticism, pleasure, intimacy, and reproduction [2]. Sexuality is experienced and expressed in thoughts, fantasies, desires, beliefs, attitudes, values, behaviors, practices, roles, and relationships. It is influenced by biological, psychological, social, economic, political, cultural, legal, historical, religious, and spiritual factors. Albeit that sexuality may include all of these dimensions, not all of them are always experienced or expressed [1].

Intellectual disability (ID) is an impairment of intellectual functioning and adaptive behavior [3]. Sexual health is strictly related to general health in both genders [4]. Disability viewed from an inclusive model, named the Social Model of Disability [5], arises from the interaction between people with impairments and barriers due to attitudes and the environment that prevent their full and effective participation in society. Becoming aware of the attitudes towards this specific population can lead to addressing the issue of health and rights to achieve inclusion since the constructed perception of women with disabilities as dispensable and dependent persons generates seclusion, a reduction of the person as a subject of rights [6].

Denial of their sexual maturity is one of the social barriers they might encounter. This barrier stems mainly from the attitudes of the “majority” society, according to which adults with ID are considered “eternal children”, even in modern times [6]. These attitudes are hard to dispute because, in society, they are not generally perceived as harmful, but rather harmless [7]. They are frequently labeled as ‘‘deviant’’ or ‘’asexual” [8]. The femininity of women with intellectual disabilities is regarded as “defective”. They cannot hold down any professional occupation and are too irresponsible to be entrusted with caring for a child [9].

The incidence of sexual violence against women with intellectual disabilities is higher than in the general population. As stated by other authors, women with any type of disability reported experiencing sexual violence in their lifetime, approximately double the proportion of that experienced by nondisabled women, with women with multiple disabilities experiencing the most significant prevalence and risk compared to non-disabled women. Women with cognitive disabilities or multiple disabilities were significantly more likely to experience either physical or nonphysical force during their first intercourse than non-disabled women [10,11,12]. Several barriers, such as deficiencies in communication and collaboration among agencies or specific training, have been described regarding sexuality in people with intellectual disabilities [13]. There are also personal factors, including those resulting from the disability itself, which are relevant. The role of environmental factors is also important. Equity in terms of the rights and experiences of the sexuality of women with intellectual disabilities is still omitted, not only because they are women, but also because of their disability [14]. Women with intellectual disabilities are subjected to discrimination against women and discrimination against persons with disabilities. They are prevented, almost without exception, from deciding for themselves about their sexual and reproductive health [15]. Usually, they are not allowed to choose a contraceptive method voluntarily or they are sterilized, as the sexual needs of women with ID have tended to be strictly controlled by their families and service workers [16].

Healthcare professionals guide patients on this important topic [4]. There is a need to develop healthcare initiatives to fulfil the requirements of families and direct care support workers regarding sexual expression in women with disabilities [17]. Some authors have described the importance of training staff to address attitudes and anxieties concerning sexuality and supporting family caregivers [18].

It is worth highlighting the need for instruments that allow for a measurement that focuses on women with intellectual disabilities and their sexuality [19]. One of the most valuable functions of a scale is the approach to prevention and decision-making, as well as its efficiency in identifying attitudes and behaviors that can be used to make decisions [20].

The importance of this issue lies in making these perceptions visible, informing and creating awareness about the aspects that we as a society can improve. In addition, the importance of making women with disabilities and their sexuality visible demonstrates the road ahead in this area since the sexuality of these women has been the subject of discussion, belonging to everyone but themselves [21]. Finally, promoting the full development of these women, the recognition of their rights and experiences, and seeking to promote the social and cultural changes necessary for a more equitable, inclusive, and just society are important [22]. Although some studies have tried to address the main barriers to expressing sexuality by adults with intellectual disabilities [17], there are few validated questionnaires developed in Spanish and tested in persons of different countries. In recent years, a great effort has been made to develop valid and reliable instruments for Spanish people with intellectual disabilities. Thus, there are self-reported and hetero-informed (parents or professionals) instruments [23].

Therefore, the main aim of this study was to design and validate a questionnaire in Spanish to acknowledge the perception of the sexuality of women with intellectual disabilities. We also sought to cover specific spheres of sexuality, such as sexual capacity, decision-making capacity, sexual disinhibition, and sexual education and quality of life.

## 2. Materials and Methods

### 2.1. Questionnaire Design

For the elaboration of the scale, fundamental issues about the sexuality of women with intellectual disabilities were considered. According to a bibliographic review, the matters chosen were the following: false beliefs [24], assumption of asexuality [8,25], or rather, a hypersexual image [26], infantilization or roles typical of girls or adolescents [9,25,27], the existence of intersectional discrimination [9,27], sterilization without prior anticipation or consultation [28,29,30], the lack of support to establish affective, loving interpersonal relationships [31,32], the lack of sex education [8,32], sociocultural barriers to live and experience their sexuality in a plane of equal rights, due to prohibitive beliefs such as the lack of control over their own lives [8,30,32,33,34], and a lack of access to health services [32,33,35,36]. Based on these topics, an initial questionnaire was developed, with 34 qualitative items and three sociodemographic questions: age, sex, and country. The responses for the items had four points on a Likert scale, eliminating the neutral option. The instrument was developed using the Google Forms platform.

### 2.2. Content Validation and Pilot Testing

A nominal group was organized, which consisted of five people from Costa Rica and seven from Spain. This nominal group was created by one of the authors of the research. Its members were chosen among experts from the two countries, and their recruitment was performed through invitations via email and phone calls. All the experts invited accepted to participate in the research, being part of the nominal group. The participation of these people was based on semantic validation, so that terms and expressions were verified. These persons were not part of the research. The specific content analysis was carried out through the supervision of eight professionals in the field of sexology and special education. In this process, each one gave their criteria, observation, correction, and scoring of the items raised, specifically valuing them as 1 = not relevant at all, 2 = not very relevant, 3 = quite relevant, and 4 = very relevant. They could also add observations and comments. The professionals made the necessary modifications to continue with the validity verification of each item using the Content Validity Index formula. This process consisted of adding the items’ points greater than or equal to 3 and dividing this by the total number of experts [37]. The questionnaire was disseminated through the use of Whatsapp and Instagram platforms. The only questionnaires admitted were those that were fully completed. Google Forms provided a spreadsheet with the responses obtained. The responses were not manipulated for statistical analysis.

### 2.3. Construct Validation

Bartlett’s sphericity tests and the Kaiser–Meyer–Olkin sampling index (KMO) were conducted to assess the exploratory factor analysis [38,39]. The KMO test statistics range from 0 to 1; those values close to 1 demonstrate higher adequacy of the factor analysis [39]. The varimax rotation method was used to include or exclude items. The varimax rotation method was chosen to maximize the weights at the factor level so that each item or variable was representative of only one to minimize the number of variables within each factor to the maximum. According to the given scores, different constructs were formed. Thus, four main domains were stated. The AMOS program carried out the confirmatory factor analysis. The maximum likelihood method was performed to validate the four constructs that had emerged from the exploratory factor analysis [40]. The goodness-of-fit index evaluates whether the confirmatory analysis fits the data [41]. In this case, the following were used: the Chi-squared test, the goodness-of-fit index, the root mean square of the approximation error, the normed fit index, the non-normed fit index, the Tucker–Lewis index, and the comparative fit index. The two-halves method was implemented since the scale could not be reperformed with those who had previously participated.

### 2.4. Statistical Analysis

The instrument underwent data analysis and the corresponding reliability measurement with IBM SPSS version 27 (IBM Inc., Armonk, NY, USA). The AMOS version 26 application (IBM Inc., Armonk, NY, USA) was used for the confirmatory analysis.

### 2.5. Ethical Aspects

This study was approved by the Research and Ethics Committee of Nursing, Physiotherapy, and Medicine Department of the University of Almeria (Spain), with Approval Number EFM 199/2022. This study used secondary data sources for the literature review and validation studies; the questionnaire did not collect personal information, and respondents’ emails were not recorded. Informed consent was shown on the initial page of the questionnaire.

## 3. Results

### 3.1. Expert Review Assessment

A total of 34 items conformed to the Likert scale. After these initial items were reviewed and rated, the Content Validity formula was applied to each item, and four items scoring less than 0.78 were eliminated. The professionals commented on the modification of words and minor changes in some questions for greater comprehension. The 12 participants of the nominal group evaluated the simplicity and semantics of the questionnaire, mentioning that it was easy to access and understand.

### 3.2. Demographic Characteristics of Participants

The total valid responses received in the Google Forms application were 244, 140 from Costa Rica (57.4%) and 104 from Spain (42.6%). The ages of the participants ranged from 18 to 74 years. The mean age was 36.1 years, with a standard deviation of 13.9. Of these, 124 (50.8%) were women and 120 (49.2%) were men.

### 3.3. Construct Validation

The result of the KMO test was 0.76, while Bartlett’s sphericity was <0.0001. The exploratory analysis excluded 20 items from the 30 items formerly assessed. The threshold used for the inclusion of factor loadings was 0.70. The analysis also identified four domains that explained 74.9% of the variance in the data. Domain 1 was measured with three items, Domain 2 with three items, the third domain with two, and the fourth domain with two, as shown in Table 1.

The domains resulting from the analysis were given a name based on their relationship. Thus, the ten resulting items were divided into four domains: sexual ability (Items 9, 10 and 12), decision-making ability (Items 23, 24, 25), sexual disinhibition (Items 1, 4), and sexual education and quality of life (Items 7, 8). Cronbach’s Alpha was 0.65 for the global questionnaire. Regarding the specific domains, Cronbach’s Alpha was 0.82 for the sexual ability domain, 0.78 for the decision-making ability domain, 0.69 for the sexual disinhibition domain, and 0.70 for the sexual education and quality of life domain. The two-halves method did not detect significant differences (Table 2).

### 3.4. Confirmatory Factorial Analysis

**Figure 1:**
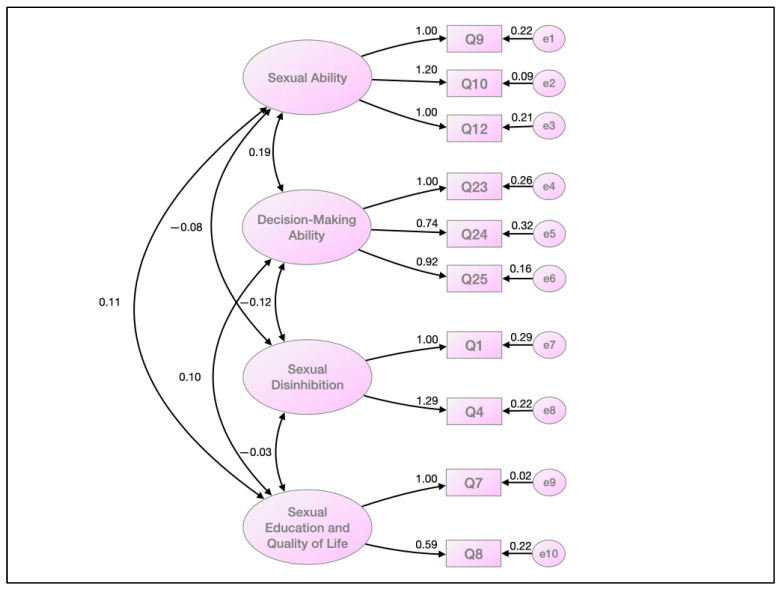
Internal measures of the construct. Confirmatory factor analysis.

The model showed 29 degrees of freedom, a Chi-squared of 39.24, and a probability level of 0.097 (Figure 1). The four constructs detected in the confirmatory analysis presented high correlations within each one of them. In addition, regression weights, covariances, correlations, and variances were calculated. The first variable, sexual ability, obtained regression values between 1.00 and 1.20. The second variable, decision-making ability capacity, ranged between 0.74 and 1.00. The third, sexual disinhibition, ranged between 1.00 and 1.29. The fourth, sexual education and quality of life, ranged between 0.59 and 1.00. The GFI value was 0.97; the NFI was 0.954; the NNFI (or TLI) was 0.98; the CFI value was 0.987, all trending toward the value of 1; the RMSEA magnitude assessment value was 0.038, with CIs of 0.001 and 0.066.

### 3.5. Resulting Proposal of the Construct Validation

After the exploratory and confirmatory factor analyses, the resulting scale items are shown in Table 3, grouped by the domains found.

## 4. Discussion

The main objective of this research was to validate a scale that can show the population’s attitudes about the sexuality of women with intellectual disabilities in different areas such as sexual ability, decision-making ability, sexual disinhibition, and sexual education and quality of life. Albeit that there are scales for the general population, no scales focus on women with disabilities [42,43,44]. There are some instruments with different versions for men and women. Other scales, although not specific for women, include aspects of female the sexuality of women with intellectual disabilities. Most scales in the literature seem not to be entirely addressed to women with intellectual disabilities, but women with physical disabilities or the male gender. Therefore, the development of our scale was essential. Even more, actual research targeting women with disabilities focuses on sexual health [16], reproductive health, and gynecological health [45]. Thus, they leave aside the broad spectrum of sexuality of women with intellectual disabilities.

We worked with 244 participants in this study to perform the validation process. As other authors recommend ten participants for each scale item [38], our study’s sample size can be considered adequate. Given the demographic aspects of the respondents, we must consider that they can be representative of the general population.

The Content Validity Index results agreed with other authors, who recommended that a scale reviewed by six to eight experts should have a minimum result of 0.83 for each item [46]. Other authors stated 0.78 as a minimum [37,47]. As a result, the items under 0.78 were discarded. Only items that scored between 0.87 and 1 were maintained to perform the exploratory and confirmatory factorial analyses. A four-point scale was used to decrease choice and ambiguity in responses.

The exploratory factor analysis excluded 20 of the 30 remaining items. Albeit that the number of questions eliminated can seem significant, it is in line with a similar number in other questionnaires [48]. Only 10 of the original 34 qualitative items remained. Therefore, only the most critical items were kept after the validation process. This removal process added value to the scale. Four main domains emerged according to what each had in common by performing the same analysis with the remaining ten items. The result of the analysis provided the model presented in this paper.

The Cronbach’s Alpha value was used to validate the questionnaire’s consistency and the coherence of the domains obtained. The overall Cronbach’s Alpha was 0.65, a value that can be considered acceptable [39,49]. The multidimensional structure of the questionnaire can lower the overall value of the Cronbach’s Alpha of the questionnaire [50]. Thus, the factorial analyses of the items were performed to group them and calculate the value of each dimension. As a result, the Cronbach’s Alpha of three of the four domains was above 0.70. Therefore, there is a high correlation between the different dimensions and their items. A set of items may be interrelated and show multidimensionality.

The confirmatory factorial analysis was performed using the maximum likelihood (ML) estimation method, the most-used method in the literature [51]. Different models were tested, and the one with the best goodness-of-fit index was the one proposed. The model’s goodness-of-fit tests were also adequate, according to the values referred to in the literature [39,52]. The RMSEA value should be less than 0.08, with a CI that does not contain it. In addition, a CFI and a TLI greater than 0.95 indicate a good model fit [53]. Internal convergent validity was performed to estimate the proportion in which the variance of the indicators of a factor was explained by an attributable source, the factor, and not by non-attributable sources, the errors.

The main limitation of this research is that the sample had a selection bias since the dissemination was through social networks, Whatsapp and Instagram, in only two countries. Although most inhabitants of both countries have access to the Internet and social networks, this recruitment method must be considered when assessing the sample representativeness of the general population. Participation was voluntary, which may have generated a self-selection bias so that the participants could have been people with greater technology access or more interest in the subject matter. These aspects should be considered when evaluating the external validity of the conclusions of this study. The research also has some strengths. The main one is that this study allowed the creation of the first questionnaire focused on the social perspective about the sexuality of women with intellectual disabilities, a tool that did not exist. An adequate number of samples were accessed, and the scale obtained good results in all validation tests, standing out in the exploratory and confirmatory factor analysis. The instrument was developed in Spanish. Various cultural or social aspects can modulate attitudes, so having scales adapted to a given context can be helpful. The questionnaire could be translated into English and culturally adapted to other countries for future research. New studies could be conducted with larger samples and different characteristics to confirm the questionnaire’s validity in these populations.

## 5. Conclusions

The final ten-item scale developed in this research proved to be a valid and reliable instrument, as it has good psychometric properties of both validity and reliability. Thus, researchers interested in investigating the social perception of the sexuality of women with intellectual disabilities can use this tool. Future research can extend the validity of this scale to other languages and settings. This research provides a new scale for quantitative studies on the sexuality of women with intellectual disabilities and an initiative for research focused on women with intellectual disabilities and their experience of sexuality. The sexuality of women with intellectual disabilities is an issue that should continue to be made visible from the rights and equity approach. It should cover topics that inform and raise awareness among the population to change social and cultural paradigms, seeking a boost to freedom, autonomy, and improvement in the quality of life of these women.

## Figures and Tables

**Table 1:** Rotated component matrix.

Item	1	2	3	4
Q1			0.87	
Q4			0.84	
Q7				0.83
Q8				0.88
Q9	0.86			
Q10	0.82			
Q23		0.83		
Q24		0.84		
Q25		0.72		
Q12	0.75			

Rotation method: varimax with Kaiser–Meyer–Olkin standardization.

**Table 2:** Two-halves test.

	n	Mean	SD *	*p*-Value **
First half	122	31.49	3.79	0.100
Second half	122	32.40	3.06	

* Standard Deviation ** U-Mann-Whitney Test.

**Table 3:** Final scale on the social attitudes of sexuality of women with disabilities.

**Domain 1. Sexual Ability**
Q9. It is challenging for women with intellectual disabilities to maintain an emotional and intimate relationship with a partner.
Q10. It is recommended that women with intellectual disabilities only engage in friendly relationships.
Q12. Marriage should not be encouraged for women with intellectual disabilities.
**Domain 2. Decision-Making Ability**
Q23. Sterilization without prior consultation with a woman with intellectual disabilities is a justifiable way to avoid unplanned pregnancies.
Q24. Women with intellectual disabilities should be involved in the decision to sterilize their bodies.
Q25. Sterilizing women with intellectual disabilities without prior consultation is fine, as the family can live without fear.
**Domain 3. Sexual Disinhibition**
Q1. Women with intellectual disabilities usually have more sexual interests than women who do not have a disabling condition.
Q4. Women with intellectual disabilities are more expressive and affectionate because they are uninhibited.
**Domain 4. Sexual Education and Quality of Life**
Q7. Sex education could improve the quality of life of women with intellectual disabilities.
Q8. Sex education could prevent abuse in women with intellectual disabilities.

## Data Availability

Not applicable.
